# Antibacterial and Anti-Inflammatory Properties of ZnO Nanoparticles Synthesized by a Green Method Using *Sargassum* Extracts

**DOI:** 10.3390/ijms24021474

**Published:** 2023-01-12

**Authors:** Jose Luis Lopez-Miranda, Gustavo A. Molina, Marlen Alexis González-Reyna, Beatriz Liliana España-Sánchez, Rodrigo Esparza, Rodolfo Silva, Miriam Estévez

**Affiliations:** 1 Centro de Física Aplicada y Tecnología Avanzada, Universidad Nacional Autónoma de México, Boulevard Juriquilla 3001, Querétaro 76230, Mexico; 2 CONACYT_Centro de Investigación y Desarrollo Tecnológico en Electroquímica SC, Parque Tecnológico Querétaro s/n Sanfandila, Pedro Escobedo 76703, Mexico; 3 Instituto de Ingeniería, Universidad Nacional Autónoma de México, Edificio 17, Ciudad Universitaria, Coyoacán 04510, Mexico

**Keywords:** ZnO nanoparticles, *sargassum* extracts, green synthesis, antibacterial activity, anti-inflammatory activity

## Abstract

The present work shows the synthesis of ZnO nanoparticles through a green method, using *sargassum* extracts, which provide the reducing and stabilizing compounds. The conditions of the medium in which the reaction was carried out was evaluated, that is, magnetic stirring, ultrasound assisted, and resting condition. UV-Vis, FTIR spectroscopy, and X-ray diffraction results confirmed the synthesis of ZnO with nanometric crystal size. The scanning electron microscopy analysis showed that the morphology and size of the particles depends on the synthesis condition used. It obtained particles between 20 and 200 nm in the sample without agitation, while the samples with stirring and ultrasound were 80 nm and 100 nm, respectively. ZnO nanoparticles showed antibacterial activity against Gram-positive *S. aureus* and Gram-negative *P. aeruginosa*. A quantitative analysis was performed by varying the concentration of ZnO nanoparticles. In all cases, the antibacterial activity against Gram-positives was greater than against Gram-negatives. Ultrasound-assisted ZnO nanoparticles showed the highest activity, around 99% and 80% for *S. aureus* and *P. aeruginosa*, respectively. Similar results were obtained in the study of the anti-inflammatory activity of ZnO nanoparticles; the ultrasound-assisted sample exhibited the highest percentage (93%), even above that shown by diclofenac, which was used as a reference. Therefore, the ZnO nanoparticles synthesized with *sargassum* extracts have properties that can be used safely and efficiently in the field of biomedicine.

## 1. Introduction

Zinc oxide is a semiconductor material that has been used in a wide range of applications [1]. In the field of biomedicine, it has been used due to its antibacterial activity and non-toxicity [2,3]. These and other properties can be improved when ZnO is at the nanometric scale. In this sense, the synthesis of nanoparticles (NPs) [4], nanowires [5], nanostars [6], and nanoflowers [7] have been reported. ZnO nanoparticles have been widely studied due to their catalytic [8], antibacterial [9], and antiviral [10] properties, which largely depend on their size and morphology. These characteristics can be controlled by varying experimental parameters, such as temperature, concentration, and rate of addition of the reagents involved.

ZnO nanoparticles have been obtained by chemical [11], physical [12] and biological methods [13]. It has been claimed that ZnO NPs can be successfully synthesized from natural sources, such as plants [14,15,16], fruits [17,18,19], and recently alga species [20,21,22]. The synergy between their size/morphology and positive surface potential can produce functional NPs with high antibacterial and anti-inflammatory features. The ZnO nanoparticles biosynthesized are of great interest, mainly when applied in areas, such as biomedicine, due to their low or null toxicity. In this synthesis, organisms are responsible for reducing and stabilizing the nanoparticles.

Algae or plants-based extracts stand out among the most used organisms since the process is simpler, reproducible, economical, and environmentally friendly [16,23]. In addition, the organic compounds from the extracts can give or increase specific properties of the nanoparticles. The use of algae for these purposes is even more attractive when these species are considered a pest. This is the case with the *sargassum* that arrives on the Mexican Caribbean coast. Each year since 2016, the amount of this algae has increased significantly because of climate change. Their overpopulation has brought severe environmental problems, such as the alteration of the photosynthesis processes of other species due to the blockage of light [24,25]. Similarly, it has also brought economic problems due to the significant decrease in tourists in the spring and summer seasons. This has led to expenses or investments for its collection, storage, and disposal [26]. Therefore, *sargassum* has recently been studied to find certain alternative uses for it, such as manufacturing paper, bricks, and fertilizers [24,27]. These algae that reach the Caribbean coast are made up of the *Fluitans* and *Natans* species [28], which have organic compounds, such as phlorotannins, flavonoids (in general phenolic compounds), and polysaccharides, that have considerable antioxidant capacity [29]. This means that these compounds can reduce metal ions. Therefore, in the present work, *sargassum*-based extracts obtained from the Mexican coast were used to synthesize ZnO nanoparticles whose antibacterial and anti-inflammatory properties were evaluated.

## 2. Results and Discussion

**Figure 1:**
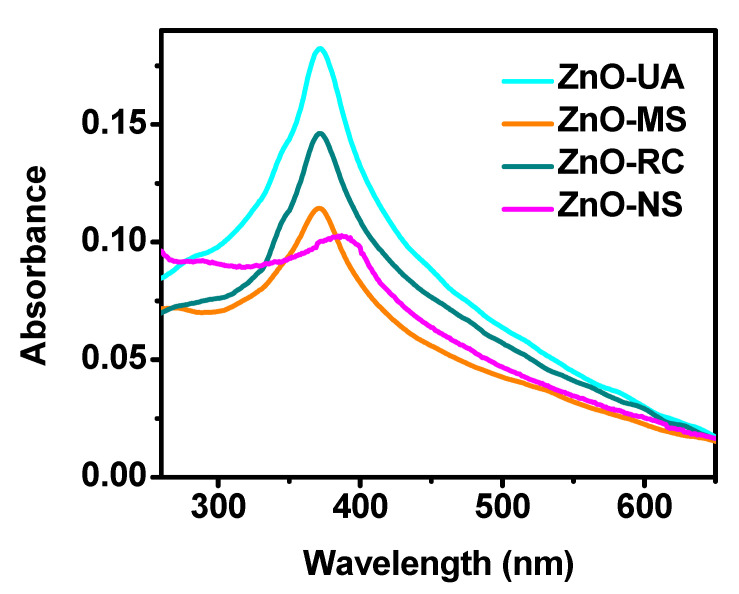
UV-Vis spectra of the ZnO nanoparticles synthesized at different conditions.

The Figure 1 shows the UV-Vis spectroscopy results of the synthesized ZnO nanoparticles. This technique is widely used in analyzing these nanoparticles since they show an absorption band between 300 and 400 nm [30,31]. The medium in which the reaction was carried out was varied. In this way, three different samples were obtained; first, the ZnO nanoparticles obtained under resting conditions (ZnO-RC), the nanoparticles synthesized with magnetic stirring (ZnO-MS), and finally, the ZnO nanoparticles assisted by ultrasound (ZnO-UA). Additionally, as a comparative reference, a sample without the addition of *sargassum* extract was prepared (ZnO-NS). The spectra corresponding to the ZnO-UA, ZnO-MS, and ZnO-RC samples show an absorption band located at 370 nm, confirming the synthesis of ZnO. The similarity in the position suggests that the average size of the nanoparticles is similar in the three samples. The spectrum of the ZnO-NS sample shows the signal at 390 nm, indicating the possible synthesis of large particles. Furthermore, the size distribution, related to the width of the band, is evidently different. The absorption band of the ZnO-UA sample is significantly more intense, suggesting a higher concentration of nanoparticles, which will be corroborated by the other characterization techniques, since the absorbance of these signals can be affected by the formation of agglomerates or the presence of other elements, such as residues of the salts used or organic compounds from the extracts.

**Figure 2:**
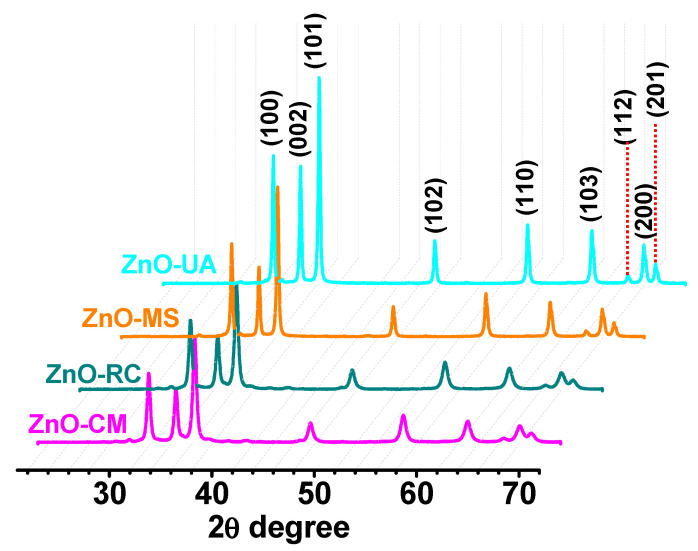
X-ray diffractograms of the ZnO samples synthesized at different conditions.

The crystalline nature of the ZnO samples was confirmed by X-ray diffraction, the results of which are shown in the Figure 2. The three samples are free of organic residues or impurities containing a single phase that was indexed with the card JCPDS 00-065-3411 card corresponding to ZnO with a hexagonal structure, P63mc space group, and lattice parameters of a = 3.2495 Å and c = 5.2069 Å. The planes correspond to the reflections located at 31.8°, 34.45°, 36.29°, 47.58°, 56.65°, 62.92°, 66.45°, 68.02°, and 69.16, corresponding to (100), (002), (101), (102), (110), (103), (200), (112), and (201), respectively. The Williamson–Hall method was used to determine the crystal size, finding values of 13.2 nm, 15.7 nm, 27.6 nm, and 25.8 nm for the ZnO-UA, ZnO-MS, ZnO-RC, and ZnO-NS samples, respectively.

**Figure 3:**
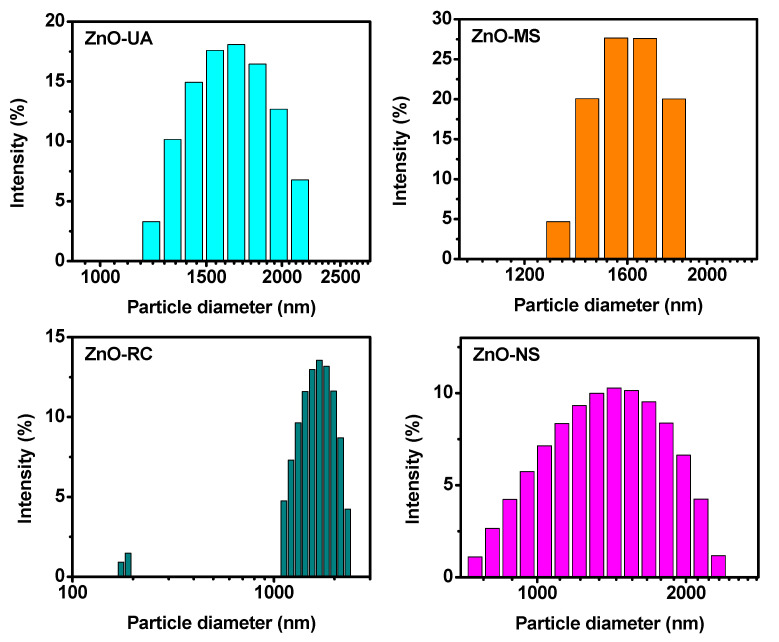
Size distribution of ZnO NPs from dynamic light scattering (DLS).

The particle size of the ZnO nanoparticles was measured by dynamic light scattering (DLS), which revealed that they were all strongly scattering samples. Then they were analyzed with a measurement angle of 175°. The particle size distribution is plotted in Figure 3 with their calculated hydrodynamic diameter (HD) and polydispersity index (PDI). Here, one element that stands out from Figure 3 is related to the number of peaks from the size distribution plot. The ZnO-UA and ZnO-MS samples show only one size distribution. The ZnO-UA peak is centered at 1695 nm, has an HD of 2950 nm, and a PDI of 18%. The ZnO-MS peak is centered at 1606 nm, with an HD of 2591 nm and 12% PDI. On the other hand, ZnO-RC and ZnO-NS present two. ZnO-RC has one centered at 183 nm and the other at 1659 nm, and ZnO-NS has one centered at 107 nm and the other at 1416 nm. Additionally, their HD is 2721 nm and 2299.91 nm, while their PDI is 27% and 29%, respectively. Those parameters suggest that the samples may have different agglomeration states. Also, the motion state affects the kinetic of the synthesis and, consequently, changes in their morphology.

**Figure 4:**
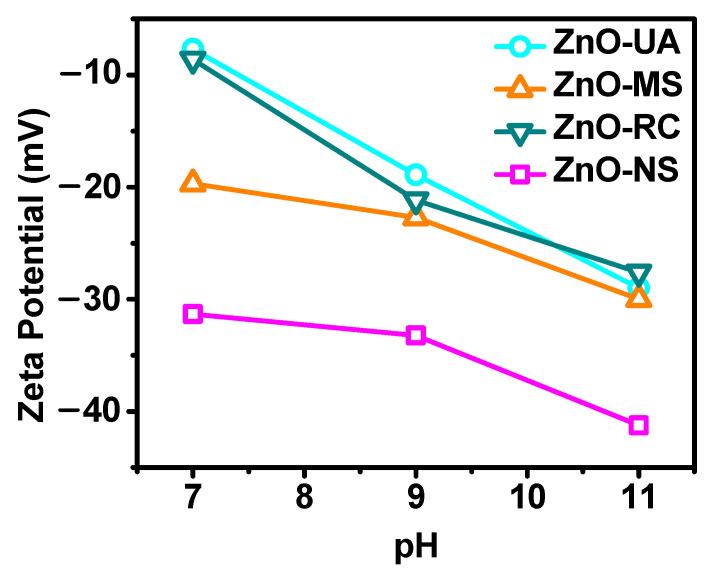
Zeta potential analysis of ZnO NPs at different pH values.

The zeta potential was performed to corroborate agglomeration, a technique that provides information about the stability of a colloidal dispersion. The resultant surface charge obtained is plotted in Figure 4. It displays how the three samples increase their colloidal stability as the pH rises. In particular, at pH 7, all the particles present a small negative zeta potential, suggesting the possible formation of agglomerates made up of nanoparticles, except by the ZnO-NS. Then, the colloidal stability, along with their negative charge, increases as the solution turns more alkaline. Here, the ZnO-MS sample is the most stable obtaining a potential of −30 eV at pH 11. Interestingly, even when at pH 7, the ZnO-MS particles have a more negative zeta potential; as the pH increases, all tend to reach similar values, suggesting different surface chemistry or even a zeta potential dependent on the morphology.

**Figure 5:**
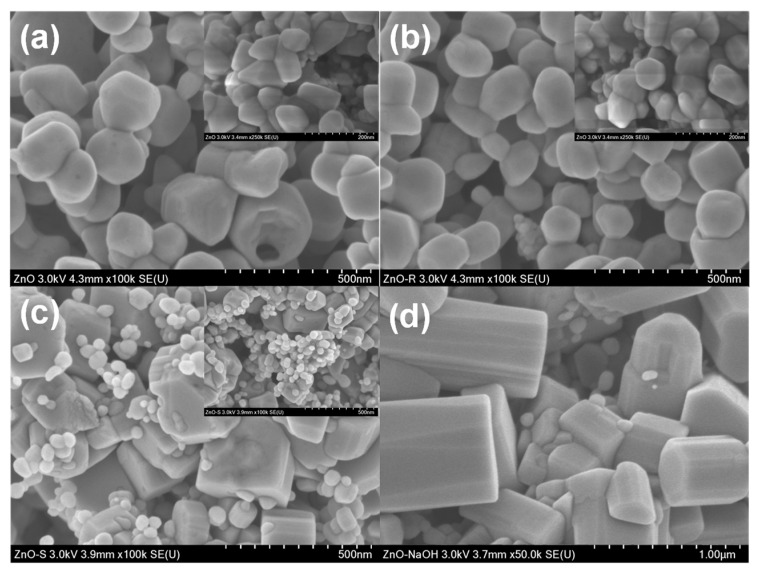
SEM analysis of the ZnO NPs obtained at different conditions: (**a**) ZnO-UA, (**b**) ZnO-MS, (**c**) ZnO-RC, and (**d**) ZnO-NS.

Figure 5 shows the analysis of the morphology and size of the nanoparticles that were evaluated by scanning electron microscopy. Figure 5a shows the nanoparticles obtained under ultrasound (ZnO-UA). The predominant morphology is irregular, with a size between 80 and 100 nm. Figure 5b corresponds to the ZnO-MS sample, where faceted particles with an irregular morphology can be seen, sizing between 100 and 150 nm. It should be noted that both samples show agglomeration, and even some coagulation. This characteristic justifies the results obtained by DLS, where the measurement corresponds to the hydrodynamic diameter, which is formed by more than one particle. Figure 5c, corresponding to the ZnO-RC sample, which shows an evident bimodal distribution. The first one is with quasi-spherical particles with a size between 20 and 50 nm. The second distribution is made up of particles larger than 200 nm, elongated with hexagonal faces that are typical of ZnO. Samples synthesized with *sargassum* extracts show a wide size distribution where a significant portion is within the nanometer scale. This can be clearly seen in the inserts of the corresponding micrographs. Even though not all the particles are smaller than 100 nm, they are frequently named as nanoparticles, as has been reported in previous works [32,33,34,35,36,37]. Finally, Figure 5d, which corresponds to the ZnO-NS sample, shows particles larger than 500 nm, with a roller-like morphology with faceted sides. This morphology has been previously reported in the absence of stabilizing agents or compounds that promote preferential growth. These results corroborate the data obtained by DLS shown in Figure 3.

**Figure 6:**
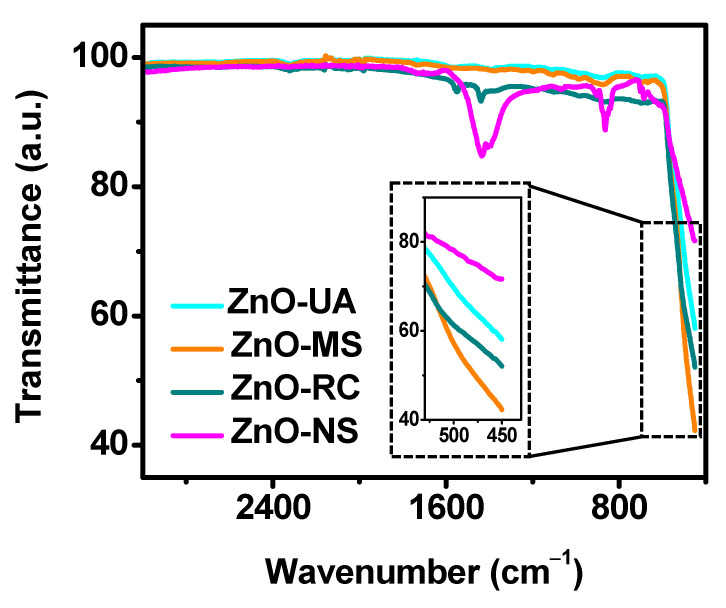
ATR-FTIR analysis of the ZnO NPs synthesized at different experimental conditions.

Different properties of the particles are related to their chemical surface; for instance, their antibacterial and anti-inflammatory activity. Therefore, ATR-FTIR analysis was performed on the ZnO samples. Figure 6 shows an almost flat spectrum obtained from all the samples, with no evidence of the presence of any hydroxide in the systems. Centered around 450 cm^−1^ and 865 cm^−1^ are an intense sharp signal and a small band due to stretching modes of ZnO [38,39]. Contrary to ZnO-MS and ZnO-UA, ZnO-RC and ZnO-NS presented additional absorption bands between 1550 and 1390 cm^−1^, ascribed to organic impurities from the Zn acetate used as a precursor.

Therefore, because all show the same chemical composition and concentration, the change in the zeta potential could be associated with their morphology, which causes variations in the surface area. ZnO-RC shows more aggregation of small and big particles, and as the pH increases, it also improves the electrostatic stabilization by increasing the repulsion forces between particles. Similar behavior is shown in ZnO-UA, where the effect on particle interaction due to the distribution of charged species in the system de-agglomerates them. Still, the zeta potential is lower than ZnO-RC because the sample only has one size distribution. The difference in the surface area caused by the smaller particles increases the pH dependency effect. On the other hand, the ZnO-MS shows the slightest variation in zeta potential against pH. Looking at the SEM micrography, ZnO-MS shows aggregation and coagulation, and according to DLS, they have smaller HD. Therefore, they have a more negative zeta potential in a neutral environment due to their minor HD. But, to disperse them, they not only need an enhanced surface ionization, provided by increasing the pH, but they may also need an externally applied force to separate them, reducing the zeta potential variations. Finally, ZnO-NS behavior suggests that the particles are stable colloids even at neutral pH, but because of their bigger size, they have low pH dependence.

The mechanism of ZnO formation through this synthesis method occurs in two stages [40,41,42]. The first consists in the formation of Zn(OH)_2_ according to Equation (1). In the sample synthesized with the absence of *sargassum* extract, (OH)^−1^ are provided by NaOH while when the extracts were used, (OH)^−1^ are provided both by NaOH and by the different bioactive organic compounds. The second part (Equation (2)) consists of the decomposition of Zn(OH)_2_ to give rise to the formation of ZnO and H_2_O, which occurs during calcination. Organic compounds from *sargassum* play a fundamental role, not only in the reduction of Zn^2+^ but also in the stabilization process, controlling the growth of ZnO, which is clearly shown in the SEM analysis.

(1) $${\mathrm{Zn}}^{2+}+{\mathrm{OH}}^{-}\to \mathrm{Zn}{\left(\mathrm{OH}\right)}_{2}$$

(2) $$\mathrm{Zn}{\left(\mathrm{OH}\right)}_{2}\overset{\Delta }{\to }\mathrm{ZnO}+H_{2}O$$

**Figure 7:**
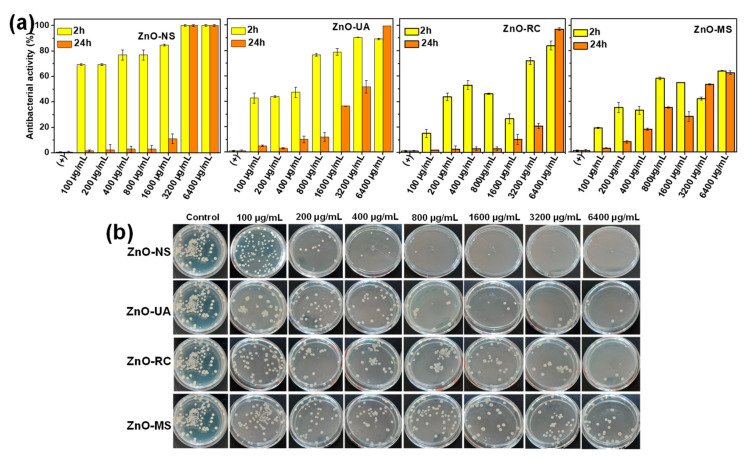
(**a**) Antibacterial activity test of ZnO nanoparticles obtained from *Sargassum* ssp. at different experimental conditions. (**b**) Representative photographs of plates obtained from the interaction of ZnO nanoparticles after 2 h of contact against Gram-positive *S. aureus*.

**Figure 8:**
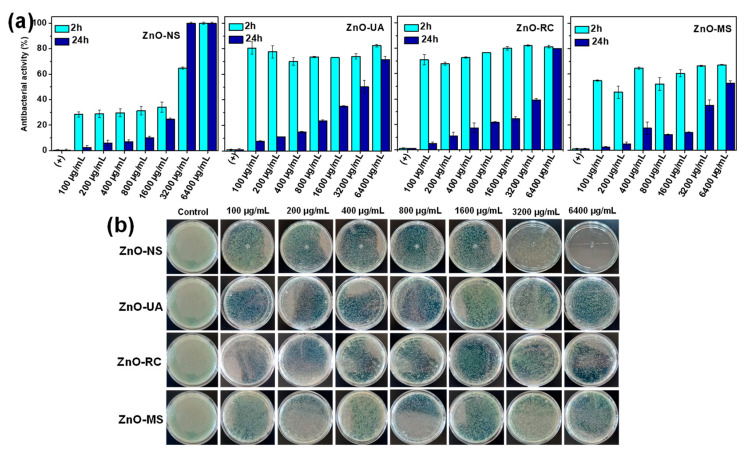
(**a**) Antibacterial activity test of ZnO nanoparticles obtained from *Sargassum* ssp. at different experimental conditions. (**b**) Representative photographs of plates obtained from the interaction of ZnO nanoparticles after 2 h of contact against Gram-negative *P. aeruginosa*.

Green synthesized NPs have gained attractive biomedical attention due to their potential biological application as antibacterial agents, avoiding the cytotoxic effects of the commonly catalytic agents. In this regard, the quantitative assays of the antibacterial activity (AA) of ZnO NPs synthesized from *Sargassum* ssp. were evaluated against Gram-positive *S. aureus* (Figure 7) and Gram-negative *P. aeruginosa* (Figure 8) and compared with the conventional ZnO NPs synthesized without *Sargassum* ssp. as reference. The ZnO NPs synthesized by conventional chemical reduction method shows an AA ca. 70–80%, with the complete bacterial inhibition in the highest NPs concentration (3200 and 6400 µg/mL), suggesting that the avoidance of the *sargassum* extract perform the complete ion release of NPs, which can be potentially detrimental under controlled ion release systems. In particular, the interaction of ZnO NPs obtained from the sonochemical method (ZnO-UA) with *S. aureus* (Figure 7a) shows an AA higher than 80% at 2 h of contact (from 800 to 6400 µg/mL) and the complete AA (99.99%) after 24 h at 6400 µg/mL. A decrease of the AA is presented in the ZnO-RC (AA ca. 40–80%) after 2 h of contact with the bacteria. However, after 24 h, null AA is observed in all NPs concentrations except at 6400 µg/mL. Finally, the AA of ZnO-MS NP is presented ca. 30–60% after 2 and 24 h of contact. This behavior suggests that the NP’s size and dispersion into the bacterial solution vary between the different synthesis methods, reflecting high AA in the ZnO nanostructures obtained from ultrasound conditions. Representative photographs of bacteria plates were presented at the bottom of Figure 7b.

In contrast, moderate AA of ZnO NPs was obtained by the interaction against Gram-negative *P. aeruginosa*, as presented in Figure 8. The highest AA is observed in ZnO-UA NPs (ca. 80%) in all NPs concentrations, demonstrating higher reactive properties associated with the smaller NPs size and dispersion by the ultrasound conditions, and compared with the ZnO-MS and ZnO-RC NPs. It is important to mention that the highest AA behavior of all ZnO NPs against *P. aeruginosa* is presented only after 2 h of contact (as seen in the bottom of Figure 8b), without AA after 24 h. The above suggests that the bacterial wall composition is key to obtaining bactericidal ZnO nanostructures.

Specific antibacterial behavior of ZnO NPs against Gram-positive microorganisms is commonly associated with the overproduction of reactive oxygen species (ROS), capable of penetrating the single peptidoglycan layer of *S. aureus*, as noted by diverse authors [14,17]. As a result, higher AA is observed at short contact time (2 h), as compared with the moderate AA of Gram-negative *P. aeruginosa*. According to Sirelkhatim et al. [43], the NPs size and morphology combined with their intrinsic photo-oxidizing and photocatalytic properties of ZnO NPs exert their antibacterial behavior. As a result, the bactericidal mechanism of ZnO NPs is attributed to the NPs internalization into the bacterial wall and the ROS formation, including species, such as hydrogen peroxide (H_2_O_2_), hydroxyl radicals (OH^–^), and peroxide (O_2_^−2^). Furthermore, it has been claimed that the ROS species perform an irreversible oxidative stress process that, combined with the Zn ions released with the ZnO NPs, can generate changes in the membrane permeability and affectations in the gene expression, generating eventual bacterial inhibition and cell death at short contact time.

**Figure 9:**
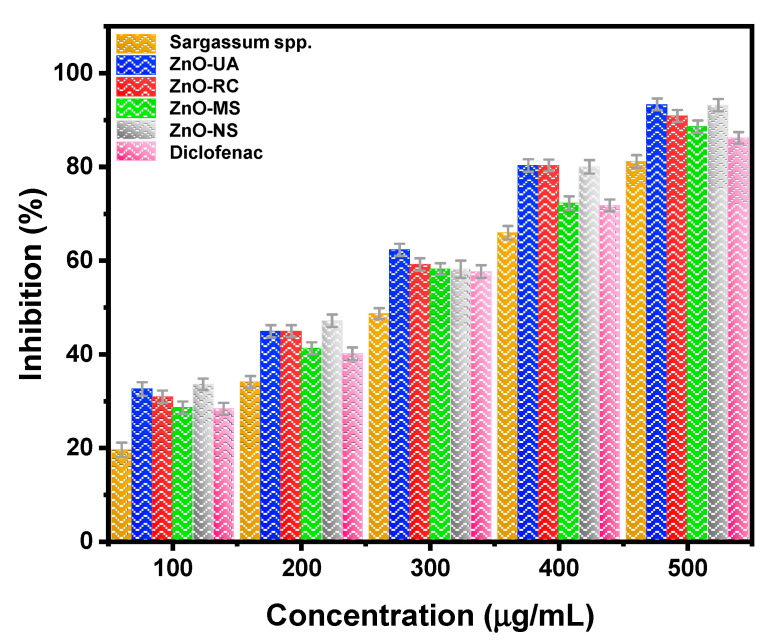
Anti-inflammatory activity of *sargassum* extract, green synthesized ZnO-UA, ZnO-RC, ZnO-MS nanoparticles, and DFS.

Sometimes it is extremely difficult to use animals for experimental research since there are ethical concerns about their use when other suitable methods can be available for analysis. As a result of this, a protein denaturation (destruction of secondary or ternary structures) assay was performed to assess the anti-inflammatory activity since this process is associated with causes of inflammatory or arthritic diseases. The increment in absorbance of the test samples to control indicated stabilization of the protein (inhibition of heat-induced denaturation) by using a model drug, the *Sargassum* spp. extract, or the ZnO NPs, is observed in Figure 9 and Table 1. This analysis is interesting for observing the ability of specific nanoparticles to protect human health or their potential use for bioactive applications. For this study, ZnO NPs can inhibit protein denaturation and, therefore, prevent/avoid the loss of biological function.

At first glance, it is observed that in all cases, as the concentration of the tested samples increases, its protein inhibition is also increased. The anti-inflammatory activity due to ZnO nanoparticles has been reported to occur in several ways, mainly due to the reduced production of thymic stromal lymphopoietin that is released by epithelial cells due to the presence of pathogenic microbes, external or internal lesions, trapped foreign particles, and inflammatory cytokines [44]. The maximum anti-inflammatory activity is obtained using the green ZnO-UA nanoparticles (93.37%), which is extremely close to the obtained value from chemically synthesized ZnO nanoparticles. As observed, all the green synthesized ZnO NPs and the chemical ZnO nanoparticles (ZnO-NS) exhibit higher (more efficient) inhibition of protein than the model drug DFS and their respective *Sargassum* spp. extract in all concentrations measured. Between green ZnO nanoparticles, it is observed that ZnO-UA has higher anti-inflammatory activity than ZnO-MS and ZnO-RC nanoparticles, which is comparable to the one obtained by the chemical synthesis method. The result indicates that the nanoparticles have a higher number of phenolic compounds capping the surface (as observed in the FTIR analysis) and, thus, a more efficient effect inhibition for protein denaturation by covalent and/or non-covalent binding in the loop 4 (domain III) of the ternary structure of albumin, especially on tryptophan, tyrosine and lysine amino acids, unlike the normal Zn(II) binding to an imidazole and then to the protein [45]. In other words, there is no intermediate molecule between the nanoparticles and the protein, and this is why with higher phenolic compounds on the surface, there is higher anti-inflammatory activity.

Additionally, the IC_50_ value was calculated for each sample to understand their effectiveness for anti-inflammatory activity better since it measures the needed quantity of the sample to achieve 50% activity. The lower the IC_50_, the higher the sample’s effectiveness because lower concentrations are required for the anti-inflammatory effect. Also, the IC_90_ values were calculated to estimate the needed quantity to achieve the full anti-inflammatory effect of the samples.

The ZnO-UA green synthesized nanoparticles show the lowest IC_50_ value (219.13 μg·mL^−1^) between green synthesis (ZnO-RC: 227.55 μg·mL^−1^ and ZnO-MS: 248.30 μg·mL^−1^), as expected since ZnO-UA has the highest inhibition percentage values. Also, it has a lower value compared to *Sargassum* spp. extract (300.63 μg·mL^−1^) and to DFS (253.73 μg·mL^−1^), but slightly higher when compared to the chemical ZnO nanoparticles (218.21 μg·mL^−1^).

When comparing our results to recent works on ZnO nanoparticles by green synthesis using the same protocol of denatured protein inhibition, it is observed that the inhibition percentage obtained is similar to the one obtained by Velsankar et al. [46] (IC_50_: 222.01 μg·mL^−1^, rectangular shaped from 15 to 30 nm) who used an *E. Variegata* leaf extract, but it is lower than the values obtained by Thaoi et al. [47] (IC_50_: 72.35 and 63.29 μg·mL^−1^, no shape reported from 40 to 50 nm) who use *Heritiera fomes* and *Sonneratia apetala* extracts, and also lower than Rajakumar et al. [48] (IC_50_: 222.01 μg·mL^−1^, spherical shape from 95 to 115 nm and hexagonal shape of 57 nm) who use *Andrographis paniculate* leaf extract. Therefore, our results indicate the potential ability of ZnO-UA nanoparticles to be used as a part of an anti-inflammatory treatment or drug.

Finally, from the IC_90_ values, the same tendency observed from IC_50_ is maintained; the samples from the green synthesis present values of 473.91 and 485.05 μg·mL^−1^ from ZnO-UA and ZnO-RC, respectively, for complete inhibition, which is within the range of the tested concentration. The IC_90_ from ZnO nanoparticles chemically synthesized is also within the concentration tested, but unlike IC_50_, more is needed compared to the ZnO-UA sample to reach the same inhibition value. For the rest of the cases, higher doses are required than those tested in the present study to obtain a complete inhibition effect, including the DFS model drug, reinforcing the green synthesized ZnO ability to protect the protein’s biological function and, thus, human health.

## 3. Materials and Methods

### 3.1. Materials

The *sargassum* was collected in Puerto Morelos, Quintana Roo (20°50′44.1′′ N, 18 86°52′35.5′′ W) in May 2022. The *sargassum* was then washed several times to remove sand and other impurities, and was then left to dry in the sun for a week and stored in the shade. Zinc acetate (99.99% trace metals basis) was used as a precursor salt, while NaOH (reagent grade, ≥ 98%, pellets, anhydrous) was used to adjust the pH of the reaction, both acquired from Sigma Aldrich. (Massachusetts, USA). Deionized water was used to prepare the corresponding solutions and dilutions.

### 3.2. Preparation of Extract

The extract was prepared placing 2 g of *sargassum* and 50 mL of deionized water in a 150 mL beaker. The mixture was heated at 60 °C, under magnetic stirring, for 30 min. Afterward, it was allowed to cool to room temperature, and the mixture was filtered using a Whatman 41 filter. The extract was refrigerated for later use.

### 3.3. Synthesis of ZnO Nanoparticles

The synthesis of ZnO nanoparticles was carried out by mixing 50 mL of an aqueous zinc acetate solution at 0.1 M with 20 mL of NaOH at 10 mM and 20 mL of *sargassum*-based extract. Three different samples were prepared by varying the medium condition during the first two hours of reaction; the first sample, named ZnO-RC, was kept at room temperature without agitation, that is, in rest condition. The sample named ZnO-MS was synthesized with magnetic stirring and, the ZnO-UA sample was assisted by an ultrasonic bath. Finally, for comparison purposes, a sample without the addition of *sargassum* extracts was prepared under the same experimental resting conditions (ZnO-NS). After, the samples remained undisturbed for 22 h. A yellowish-white precipitate was observed in the samples corresponding to the synthesized ZnO. Then, the samples were washed to remove the remaining extract and residues of the salts used. This consisted of removing the supernatant, adding deionized water, redispersing the solid, and precipitating it by centrifugation. This process was repeated three times to obtain purified ZnO. The samples were dried in an oven at 60 °C and subsequently calcined at 600 °C for 4 h.

### 3.4. Characterization

Different characterization techniques evaluated the characteristics and composition of the ZnO nanoparticles. The presence of the nanoparticles was determined by UV-Vis spectroscopy using a Metash 6000 M spectrophotometer (Shanghai, China). The analysis was carried out by diluting the nanoparticles in deionized water using quartz cells. The crystalline nature, composition, and crystal size of the samples were evaluated by X-ray diffraction using a Rigaku Ultima IV diffractometer (Tokyo, Japan). The analysis was performed in a 2θ range from 20 to 80° using Cu Kα radiation. The particle size and stability were estimated by dynamic light scattering (DLS) using a Litesizer 500 Anton Paar (Graz, Austria) particle analyzer, with a semiconductor laser diode (λ = 658 nm) and a disposable cell. The zeta potential analysis was conducted to evaluate the colloidal stability of the samples using the Litesizer 500 and reusable cuvettes. The morphology and size of the ZnO nanoparticles were analyzed using a cold-field emission scanning electron microscope (Hitachi SU8230, Tokyo Japan). The organic compounds involved in the reduction and stabilization were suggested based on the results of FTIR spectroscopy performed on a Perkin Elmer, Spectrum Two (Massachusetts, USA).

### 3.5. Antibacterial Activity

Antibacterial activity (AA) was performed against Gram-positive *Staphylococcus aureus* ATCC #6538 and Gram-negative *Pseudomonas aeruginosa* ATCC #13388, according to the reported in our previous works [49,50]. Briefly, inoculums of each microorganism were grown in Luria Bertani broth (LB) at 36 °C for 16 h and adjusted to a final concentration of 1 × 106 unit forming colonies (UFC)/mL. ZnO nanoparticles concentrations (100, 200, 400, 800, 1600, 3200, and 6400 µg/mL) were suspended in sterile phosphate buffer (PBS) with Tween 80 at 1 wt. % as a dispersant agent to promote the NPs dispersion. Each NP’s suspension interacted 1:1 with the bacterial inoculum during 2 and 24 h at 37 °C. After NPs/bacteria interaction, an aliquot of 50 µL of the bacterial recovery was plated in LB dishes and incubated for 16 h at 37 °C. The AA evaluation was performed by three independent experiments by duplicated.

### 3.6. Evaluation of Anti-Inflammatory Properties

The Chandra et al. procedure [51] for in-vitro anti-inflammatory activity was followed for measuring the different ZnO nanoparticles (ZnO-NPs). First, a 2 mL solution of the tested samples (extract or ZnO-NPs) was prepared at different concentrations: 100, 200, 300, 400, and 500 μg/mL. Then, a 5 mL solution was prepared using the 2 mL samples mixed with 0.2 mL of egg albumin (Huevo San Juan, San Juan de Los Lagos, Jalisco, México) and 2.8 mL of phosphate buffer (PBS, pH 6.4). Solutions at the same concentrations using diclofenac sodium salt (DFS) were used as a reference (positive control), and a solution with 2 mL of deionized water served as a control. All solutions were homogenized using a vortex agitator.

The prepared solutions were incubated at 37 + 2 °C using a lab-made incubator for fifteen minutes, and after the elapsed time, the reaction mixture was heated up to 70 °C for five more minutes. The samples were left to stand at room temperature, and after cooling time, the solutions were measured using UV-Vis spectroscopy at a wavelength of 660 nm.

All the analyses were performed in triplicate and changes in absorbance at 660 nm were noted, and the percentage of inhibition of protein denaturation was calculated using the equation:

(3) $$Inhibition\;\left(\%\right)=\left(\frac{A_{C}-A_{S}}{A_{C}}\right)\times 100$$

where *A_C_* is the absorbance of the control and *A_S_* is the absorbance of the sample.

## 4. Conclusions

The results of this work demonstrated the ability of *sargassum* aqueous extracts to reduce and subsequently stabilize ZnO nanoparticles under three different conditions: rest, magnetic agitation, and ultrasound assistance. This parameter plays a fundamental role in the size, morphology, and distribution of the nanoparticles. When the synthesis was performed with ultrasound and magnetic stirring, the obtained nanoparticles were irregular in shape with a uniform distribution and an average size of 100 nm and 80 nm, respectively. The sample synthesized in resting conditions showed nanoparticles of various morphologies of sizes ranging from 20 nm to 200 nm.

Furthermore, the ZnO nanoparticles synthesized under the different conditions showed significant antibacterial activity, with the ultrasound-assisted sample having the highest percentage against *S. aureus* (99%) and *P. aeruginosa* (80%). High antibacterial activity is attributed to the differences of the bacterial wall composition, obtaining high bactericidal behavior against Gram-positive microorganisms. The same sample showed the highest anti-inflammatory activity, which was 93%, being higher than the reference drug, which in this case was diclofenac. Therefore, these ZnO nanoparticles, which were obtained by a simple, economical, and environmentally friendly method, can be used in biomedical applications in a safe, efficient, and non-toxic way.

## Figures and Tables

**Table 1:** Percentage of protein denaturation inhibition from extract, all synthesized ZnO nanoparticles, and DFS as a positive control.

Concentration (μg·mL^−1^)	Inhibition (%)					
	*Sargassum* spp.	ZnO-UA	ZnO-RC	ZnO-MS	ZnO-NS	DFS
100	19.58	32.57	30.93	28.62	33.59	28.35
200	34.10	44.92	44.93	41.30	47.18	40.10
300	48.70	62.31	59.20	58.22	58.19	57.65
400	66.00	80.33	80.33	72.30	80.03	71.80
500	81.20	93.37	90.90	88.60	93.19	86.21
Concentration (μg·mL^−1^)						
IC_50_	300.63	219.13	227.55	248.30	218.21	253.73
IC_90_	558.56	473.91	485.05	513.22	481.28	525.06

## Data Availability

Not applicable.
